# Unravelling the roadblocks and pathways to adolescents’ physical activity

**DOI:** 10.1080/17482631.2025.2524270

**Published:** 2025-07-01

**Authors:** Christina Alexandrou, Anna-Karin Lindqvist, Hanna Wieslander, Stina Rutberg

**Affiliations:** a Department of Medicine, Huddinge, Karolinska Institutet, Sweden; b Department of Health, Education and Technology, Division of Health, Medicine and Rehabilitation, Luleå University of Technology, Luleå, Sweden

**Keywords:** Physical activity, adolescents, socioeconomic factors, COVID-19 pandemic, post-pandemic

## Abstract

**Background:**

The COVID-19 pandemic negatively affected adolescents’ opportunities for physical activity in many countries.

**Aim:**

To explore experiences and opportunities for physical activity among Swedish adolescents with different backgrounds, considering both the impact of the COVID-19 pandemic and broader influencing factors during and after this period.

**Methods:**

Data was collected through focus group interviews (*n* = 13) with 86 students and analysed using content analysis.

**Results:**

Although many adolescents managed to remain physically active during the COVID-19 pandemic, socioeconomic differences affected opportunities for participation in organized sports and activities, as well as perceptions of community safety. Findings also highlighted the important role of schools and the influence of gender and performance norms on physical activity, as well as young girls’ desire for “safe spaces” to focus on their training and development.

**Conclusion:**

The COVID-19 pandemic disrupted routines, prompting adaptations and exposing various barriers. Social belonging, socioeconomic background, and prevailing norms significantly influenced activity levels, while the balance between independence and safety emerged as a key factor. Moving forward, we recommend investing in community safety, youth sports, and supportive school policies that challenge gender and performance norms, particularly in socioeconomically disadvantaged areas.

## Background

Physical activity is associated with increased physical fitness and cardiometabolic health and is protective against non-communicable diseases such as cardiovascular disease, diabetes type 2 and certain types of cancer, both directly but also indirectly by helping maintain a healthy weight (Bull et al., 2020). It also has a positive effect on mental health and wellbeing, helps reduce stress and anxiety symptoms and improve cognitive function (Bull et al., 2020). The World Health Organization recommends at least one hour of moderate to vigorous physical activity per day for adolescents (Bull et al., 2020), however, globally, most adolescents (81%) do not reach this recommendation (Guthold et al., 2020). In Sweden, only 13% and 20% of 15-year-old girls and boys respectively meet the recommendation (The Public Health Agency of Sweden, 2024a), and almost half of all 15-year-olds (44%) spend all, or almost all their leisure time sedentary (The Public Health Agency of Sweden, 2024a). Furthermore, adolescents from socioeconomically vulnerable families have less opportunities for participation in organized sports and activities (Falese et al., 2021; Tandon et al., 2021) and boys are often more active than girls (Guthold et al., 2020; The Public Health Agency of Sweden, 2024a; van Sluijs Emf et al., 2021). Clearly, more knowledge is needed about the prerequisites for being physically active across the adolescent years.

During the COVID-19 pandemic, adolescents’ opportunities for physical activity decreased in many countries, mainly due to the lockdown measures that led to the closure of community settings like schools and kindergartens (Bozzola et al., 2023; Ludwig-Walz et al., 2023). Compared to other countries, Sweden adopted a different approach, keeping kindergartens, primary and secondary schools open (Björkman et al., 2023), with some exceptions for adolescents, where remote teaching was practiced for a couple of weeks during the worst disease spread (Hall et al., 2024). Furthermore, Sweden did not enforce any strict measures regarding outdoor spaces, which allowed many sports associations to adapt and move their training outdoors (Björkman et al., 2023). As a result, there is a need for more information on how the COVID-19 pandemic affected adolescents’ (13–18 years) physical activity, and if there were any differences depending on socioeconomic factors. The Swedish Public Health Agency has already investigated young people’s (16–29 years) living conditions and health during the pandemic (The Public Health Agency of Sweden, 2024), where results showed that for young people already living in socioeconomically vulnerable situations, the pandemic interacted negatively with the existing life situation and sometimes reinforced existing challenges (The Public Health Agency of Sweden, 2024c). Thus, this study aimed to explore experiences and opportunities for physical activity among Swedish adolescents with different backgrounds, considering both the impact of the COVID-19 pandemic and broader influencing factors during and after this period.

## Methods

### Study design

The study utilized a qualitative study design (Flick, 2022) and was conducted through focus group interviews. Focus groups were chosen to facilitate interaction and discussion among youths, encouraging them to share, compare, and elaborate on their experiences. This dynamic contributed to a rich and nuanced material. The study followed the Consolidated Criteria for Reporting Qualitative Research (COREQ) checklist (Tong et al., 2007).

### Participants and recruitment

#### Schools

Purposive sampling was used to recruit schools from different socioeconomic areas in Stockholm (urban area) and Halland (rural area). The selection of high schools (i.e., grades 7 through 9) was based on their classification in the “Swedish National Agency for Education’s socioeconomic index in 2022” (Swedish National Agency for Education, 2024), which is an index predicated on the expected percentage of students, ineligible for upper secondary school. Invitations were sent out to principals and school administrations of high schools with a low to middle index (*n* = 32) and to high schools with a higher index (*n* = 5). As there currently is no similar socioeconomic index for upper-secondary schools (i.e., grades 10 through 12) in Sweden, an invitation was sent out to upper-secondary schools (*n* = 22) located in the same areas as the invited high schools. Schools that expressed interest in participating (*n* = 9) were contacted in August 2023 to schedule an information visit. In total, seven schools participated in the study: four high-schools and two upper-secondary schools in the region of Stockholm, and one high-school in region Halland.

#### Youth

Students were recruited through informational visits to the schools where they received both written and verbal information about the study procedures from CA (first author and female researcher with previous experience of qualitative data collection and analysis). Eligibility criteria included being aged between 13 and 18, willingness to share personal experiences and provision of written informed consent to participate in a focus group interview at the school, together with other students. For students younger than 15 years, written informed consent was obtained from both their caregivers and themselves, whereas students aged 15 or older provided their own written informed consent to participation prior to the interview. Language was not an exclusion criterion. In total, 87 students participated and all but one were divided into 13 focus groups (Table I). The remaining student participated in an individual interview conducted together with an Arabic speaking interpreter. The participating students were on average 15 years old, 50% were girls and 46% were foreign-born (i.e., either born abroad or had two foreign-born parents).

**Table I.** Focus group characteristics. In total, 87 students (50% girls; 46% foreign-born^a^) participated in the study through 13 focus groups and one individual interview.^b^

	High school^c^/ Upper secondary school	Socioeconomic index^d^	Mean age (years)	Group size (n)
Focus group 1	High school	Low	14.2	6
Focus group 2	High school	Middle	13.9	7
Focus group 3	High school	Middle	14.3	6
Focus group 4	Upper secondary school	Low/Middle	18.4	5
Focus group 5	High school	Low	15	6
Focus group 6	High school	Low	15.1	8
Focus group 7	High school	High	14	7
Focus group 8	High school	Middle	15	6
Focus group 9	High school	Low	14	7
Focus group 10	High school	Middle	15	7
Focus group 11	High school	Low/Middle	14	4
Focus group 12	High school	High	14	8
Focus group 13	High school	High	14	9

^a^Foreign-born, i.e., either born abroad or had two foreign-born parents.

^b^The individual interview was conducted at an upper-secondary school located in a high SEI area in Stockholm.

^c^In Sweden, high schools correspond to grades 7–9, while upper secondary schools correspond to grades 10–12.

^d^Data on socioeconomic status was collected on the school level, and not on student level. Schools were classified based on their score in the “Swedish National Agency for Education’s socioeconomic index in 2022”, which is an index predicated on the expected percentage of students, ineligible for upper secondary school.

### Data collection

Data was collected through focus group interviews (*n* = 13) and one individual interview by CA and HW, both female researchers with previous experience of qualitative research and focus group interviews. Prior to each focus group, students answered a questionnaire with demographic questions. A semi-structured interview guide (Supplementary file 1) was used to capture relevant and in-depth data. The questions focused on students’ opportunities for physical activity and participation in leisure activities during and after the COVID-19 pandemic. The interviews were performed at the premises of each participating school and ranged from 50 to 121 minutes (average 80 minutes). All interviews were audio recorded and transcribed verbatim by an external transcribing firm.

### Data analysis

After each focus group interview, CA and HW discussed initial impressions and interpretations. Based on these reflections, CA formulated a preliminary, holistic interpretation of the participants’ expressed experiences—referred to here as a naïve understanding. This initial interpretation aimed to capture an overall sense of meaning conveyed in the interviews and was later compared with the emerging categories to ensure depth and consistency in the analysis. The transcripts were analysed by CA using inductive qualitative content analysis inspired by Graneheim and Lundman (Graneheim & Lundman, 2004). The manifest content of the transcripts was first read through and meaning units answering the study aim were identified and highlighted. These meaning units were then assigned codes and compared in terms of similarities and differences. The codes were grouped into preliminary categories that reflected patterns in the data. In the next phase, we moved from manifest to latent analysis, inspired by Graneheim, Lindgren and Lundman (Graneheim et al., 2017). We examined the preliminary categories to identify underlying meanings and unifying threads, which were then formulated into sub-themes. Finally, we abstracted a main theme that captured the overarching meaning of the participants´ experiences. The drafts of sub-themes and main themes were during this process discussed together with SR and AK-L, both female researchers with extensive qualitative research experience, until consensus was reached. Two of the researchers in this study are physiotherapists (SR and AK-L) and two are nutritionists (CA and HW), all with deep expertise in behaviour change regarding physical activity.

### Ethical considerations

All students received verbal and written information about the study and their right to withdraw their participation at any timepoint, before providing written informed consent. As compensation for their participation, students received a movie ticket. The study was approved by the Swedish Ethical Review Authority in April 2023 (ref no. 2023–01085–01).

## Results

**Figure 1.**
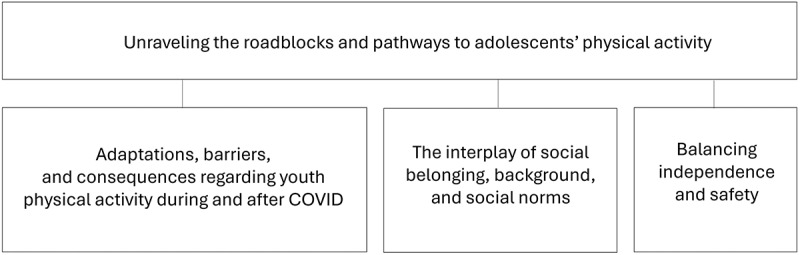
Overview of main theme and sub-themes.

The analyses identified one main theme based on three sub-themes (Figure 1). Excerpts from the interviews were included to illustrate the findings. The quotations are denoted with focus group number (no. 1–13), area (urban/rural), socioeconomic index (SEI: low, medium, high), and where relevant, with gender (girl/boy).

### Unraveling the roadblocks and pathways to adolescents’ physical activity

The findings reveal the impact of societal and personal factors on the physical activity of Swedish youth during and after the pandemic. The pandemic significantly disrupted regular physical activity routines, leading to various adaptations, barriers, and consequences. Some barriers persisted even after restrictions were lifted, continuing to hinder physical activity. Schools and teachers emerged as crucial influencers, either promoting or obstructing physical activity based on the support and opportunities they provided. Additionally, the interplay of social belonging, socioeconomic background, and social norms surrounding physical activity played a significant role in either encouraging or discouraging youth participation. Community safety, and youth’s ability to balance independence and safety, was another critical factor affecting physical activity, especially for youth in socioeconomically vulnerable areas. Unravelling the roadblocks and pathways to adolescents’ physical activity reveals a complex interplay of social, environmental and personal factors embedded in their everyday lives.

#### Adaptations, barriers, and consequences regarding youth physical activity during and after COVID

During the pandemic, Swedish youth experienced changes in their physical activity levels. Some managed to remain active by adapting to online or outdoor activities, while others became less active due to paused activities and reduced amount of active transportation to and from activities. The pandemic also negatively influenced mental health and motivation, resulting in increased reluctance to engage in activities outside the home. This decline in mental well-being often resulted in feelings of anxiety and depression, which further discouraged physical activity. Consequently, many youths adopted more sedentary lifestyles, accompanied by feelings of guilt of not being more active. While online schooling was convenient short-term, it was not preferred long-term. Additionally, the popularity of online gaming and activities like E-sports and TikTok, contributed to a more sedentary lifestyle.

**(Youth 1)** And then there’s also this lack of movement, it can make you more easily depressed and really down and just [make you] stay at home and not do anything.

**(Youth 2)** I was also going to say that you had to limit yourself a bit during the pandemic, but you mustn’t limit yourself too much. In a way, I think it’s very good that Sweden didn’t completely lock down, because you have to be out and about, you have to do some physical activity. Because if you just stay indoors, it can lead to depression and stuff like that.

**Focus group no. 12, urban area, high SEI**

**(Youth 1)** I didn’t feel much difference from before and during the pandemic

**(Youth 2)** There were more people who joined outdoor sports from other things that were cancelled, so there were more people in the team

**Focus group no. 3, rural area, mixed SEI**

After the pandemic, some youth initially felt insecure about returning to training, while others experienced fatigue and preferred staying indoors, leading to a more sedentary lifestyle. Some expressed having lost their motivation to become more active again and had adapted to a sedentary routine. Conversely, others felt more engaged in physical activities due to new interests and the natural progression of getting older. As a consequence of avoiding crowded public transport, some youth adopted more active modes of transportation, a habit that persisted post-pandemic for some.

Financial limitations post-pandemic was a barrier for youths in socioeconomically vulnerable areas to return to previous leisure activities. Increased training fees, equipment costs, and parents who had lost their job during the pandemic contributed to this barrier. They also felt excluded from sports halls as these were reserved by different sports associations. Although initiatives such as youth centres that were free of charge existed, youths emphasized the need for more such initiatives. Youths also discussed the political responsibility to invest in supportive environments, especially in disadvantaged areas. Currently, they perceived that this was not prioritized. Youths from more affluent areas also discussed how unfair it was to expect financially disadvantaged adolescents to be responsible for their own physical activity, when not having the financial means to join an activity, especially during the colder seasons.

We wanted to start training together… then we saw that it costs SEK 2,500 [260 Euro] per semester, it’s not possible. I think training should be free of charge. Because if you want kids to learn, if you want kids to be physically active, then it should be free. If you have to pay 2,500 for it, then parents might not be able to afford it.

**Focus group no. 9, urban area, low SEI**

Both during and after the pandemic schools were described as a sedentary setting, with short breaks, leaving little room for activities, and youth suggested longer breaks to encourage physical activity. As many students stayed indoors regardless of break length, youth also discussed the importance of structuring breaks to support and encourage movement. Overcrowded schoolyards also posed a barrier for spontaneous activities.

**(Youth 1)** - You spend 70% of the time in your life at school in a classroom, at a desk with a pencil. So, you usually sit for a long period. For example, in a week, how many hours of school do we have?

**(Youth 2)** Eight

**(Youth 1)** - Exactly. Every five days, there will be a lot of sitting

**Focus group no. 5, urban area, low SEI**

Long school days and schedule conflicts also dampened youths’ motivation for physical activity. In addition to this, youths expressed a need for schools to provide simple snacks to maintain focus and energy. They argued that school-provided snacks could prevent poor choices like skipping training or overeating. Despite knowing that movement promotes learning, not all teachers planned active lessons, and youths emphasized the need for enabling and supporting teachers to have more active classes, such as standing during group work, taking short walks, or other ways of increasing physical activity.

**(Youth 1)** − Most of us can’t sit still … 

**(Youth 2)** “- It becomes very difficult if you have too long lessons or too many school hours, you get quite tired”

**(Youth 3)** - Yes, but the teachers have solved that. The teachers have said that we can take a mini break in the middle of the lesson. Especially if you have difficulties concentrating, you can take mini breaks. So, there is nothing to worry about. I think it helps, because I have difficulties myself, so it helps me a lot. But the best would be to educate the teachers better [about active classes].

**(Youth 2)** − Yes.

**Focus group no. 6, urban area, low SEI**

In order to promote physical activity, the school environment was also described as ideal for informing students about local activities. Furthermore, youths felt that community planning often prioritized younger children and suggested the need for more and improved outdoor gyms and adolescent-friendly parks. Free public transport was also suggested to encourage spontaneous physical activity.

### The interplay of social belonging, background, and social norms

The interplay of social belonging, background, and social norms influenced adolescents’ engagement in physical activity. Being part of the local youth community played a crucial role in adolescent’s participation in physical activity. Inclusive spots like football fields and youth centres, fostered a sense of community and security, making spontaneous activity more accessible. Participation in organized activities or being part of a team was also valued, as it not only strengthened social connections and expanded friend networks but also increased resilience against negative influences like organized crime.

You feel safe with each other … For example, here in [the municipality] we have a big, big football field. All of us grew up with each other so we know each other, and we have our community and friends and maybe a common sport/activity at the football field. In the evenings, for example, we usually go down there and meet a lot of people from [the municipality], and me and my friends usually play matches together. So that’s something that always makes it better, having a community.

**Focus group no. 5, urban area, low SEI**

Youths’ physical activity was influenced by their sense of inclusion and acceptance in sports and activities, which in turn was affected by social norms. Youths discussed how performance norms could act as obstacles, making it both challenging and sensitive to switch activities during adolescence due to fear of lagging behind peers. Many youths expressed sticking to one activity throughout childhood, as they felt it was the only thing they could do. Gender norms labelled certain activities as “girls” or “boys” activities, like for example dance being a girl activity, but many youths ignored this when choosing activities. In addition, the use of more inclusive language and more trans youth participating in sports like football were mentioned as positive developments that also were necessary to increase opportunities for all to be included.

Social norms on weight and body image, amplified by social media, were discussed to affect some youth’s participation in physical activity negatively. Although social media could be inspiring and motivating, it could also harm self-confidence through comparison. Youths understood that being overweight could be a barrier to physical activity due to weight stigmatization and brought up examples of witnessed discrimination by both coaches and teammates. Despite initiatives like the “body positive” movement, societal norms of thinness were hard to ignore, and girls expressed that they faced more unrealistic body and weight ideals compared to boys, often requiring invasive procedures.

I had a friend who trained, and he was very overweight. Everyone teased him, saying ‘But you are fat, where are you going to run? The coach also said, “You can’t run, just stay on the bench, we will call on you”. They never called him. There are a lot of things like that.

**Focus group no. 4, urban area, mixed SEI**

Youths with immigrant background shared experiences of racialization and discrimination in physical activities, sometimes involving coaches. They felt the need to adapt their behaviour, for example the way they talked or behaved, and they sometimes faced offensive comments during matches. Some blended into their teams, while others quitted due to discrimination. Additionally, girls with immigrant backgrounds discussed how cultural norms, such as the belief that “girls should stay at home” or “not exercise and become strong” influenced their physical activity. Nevertheless, girls had also noticed a slight shift for the better regarding these cultural norms during the last couple of years.

**(Youth 1)** We usually face Swedish teams. Every time we go to them [for a match] and they see someone wearing a head covering scarf, they say, “You have bombs under your scarf” and stuff. As soon as you tell the judge, he says “No, I didn’t hear” or something’.

**(Youth 2)** I think it was [team xx] we met. She’s running, she’s behind me, she’s pulling my scarf, I feel it. The judge, he stood so he could see, but he said nothing.

**Focus group no. 9, urban area, low SEI**

Youths suggested promoting acceptance and inclusivity in sports and activities, by involving different stakeholders, like team members, coaches, parents, and judges. Positive confirmation of achievements and respectful feedback were important for self-confidence and motivation. They emphasized the careful recruitment of coaches and taking complaints about discriminatory behaviour seriously. They also discussed the importance of age-appropriate beginner groups for trying out new activities, especially during adolescence, and suggested creating a digital platform for finding and initiating beginner groups.

### Balancing independence and safety

Youths’ perceptions of community safety played a crucial role in balancing their independence and engagement in physical activity. Feeling safe in their neighbourhood influenced their willingness to move freely, whether walking, cycling, or participating in spontaneous outdoor activities. However, as they gained more independence during adolescence, many reported feeling increasingly unsafe outdoors. Fear and discomfort were particularly associated with walking or cycling through poorly lit areas. Girls felt more exposed to risk outdoors and were taught to be alert from a young age. They felt safer when other girls or women were present, creating an unspoken pact of protection. Girls also discussed how restrictions during the pandemic made outdoor areas feel safer for girls when walking or running errands alone.

“It still has a lot to do with physical activity. Because you feel insecure when you go and exercise for your health … it affects that a lot. If I feel insecure when I go somewhere, then automatically I just want to stay at home … I don’t dare go out. It shouldn’t be like this in society in general, you should be able to go and do things without feeling worried and thinking about ‘What will happen? Will someone kidnap me? Will I get shot? Is this going to happen?’ You think a lot about these things in general.”

**Focus group no. 9, young girl, urban area, low SEI**

Youths suggested several measures to enhance community safety, emphasizing the need for more night-open places to seek shelter when feeling unsafe. Early closure of public places, like youth centres, stores, and restaurants, negatively impacted their perceived safety. Another suggestion was for means of public transport to also run late on weekday evenings, and not only on weekends. In vulnerable areas, youth stressed the need for increased security personnel presence, especially during late evenings and early mornings. However, they also underlined the importance of balance to avoid a sense of “police state”. Opinions varied on the use of surveillance cameras, with some seeing them as a security enhancer, while others viewed them as providing false security if a perpetrator, for example, was masked.

Girls discussed how important it was to feel safe and comfortable to go and exercise or focus on their own exercise in gyms. They found mixed-gender gyms uncomfortable and preferred exercising in women-only spaces. They also desired more separate gym sections for women. Boys acknowledged girls’ discomfort and also felt insecure in male-dominated gyms. Both genders desired more inclusive gyms for fitness, not just for those already fit. Additionally, within the school setting, girls discussed the possibility of separate physical education classes, as they perceived boys’ choices being prioritized and thus dictating the sports and activities during classes. Boys were also considered to be more aggressive when playing in mixed teams, which limited girls and hindered them from fully developing their fitness and sports skills.

**(Youth 1, girl)** I think it’s good [with separate women’s area in the gym]. Because usually as a girl you can feel uncomfortable working out among lots of men.

**(Youth 2, boy)** Because people are checking and staring.”

**(Youth 3, boy)** I also think it’s good, because if it’s divided, then the girls… you also notice when you go to the gym, girls feel insecure, because there are still boys everywhere. I for one think that guys can’t feel the way girls feel. So, it’s better if you split it up, so girls can train what they want to train without feeling that “I have eyes on me from guys

**(Youth 1, girl)** Yes, when a lot of men look at you and sit and stare, then you become very uncomfortable, and then you just walk away.

**Focus group 4, urban area, mixed SEI**

## Discussion

The current study explored experiences and opportunities for physical activity among Swedish youth from different socioeconomic areas, during and after the COVID-19 pandemic. The findings highlight that while many Swedish youths were able to remain physically active during the pandemic, their experiences varied depending on various factors. Youth described how they adapted to the changes in their everyday lives, the barriers they encountered and the consequences these had on their physical activity patterns and routines. Findings also emphasize the impact of social belonging, socioeconomic background, and social norms in either encouraging or discouraging youth participation in physical activity. Furthermore, the necessity to balance feelings of independence and safety is emphasized, with community safety highlighted as a critical factor for youth participation in physical activities. Finally, the findings also address the challenges encountered by youth in socioeconomically disadvantaged areas. Together, these findings underscore the intricate interplay of societal and personal factors and the need for comprehensive solutions to encourage physical activity among youth.

In contrast to international reports of decreased physical activity among adolescents during the pandemic (Bozzola et al., 2023; Rossi et al., 2021), participants in the current study described having opportunities to remain physically active. Many youths reported maintaining or adapting their routines or finding new ways to be active, despite disruptions. These findings align with results from a study on Swedish 11–13-year-olds (*n* = 1103; birth-cohort subsample) where a majority reported either unchanged (42.1%), with some reporting being more active (33.9%) levels of leisure-time physical activity during the pandemic, while a smaller proportion reported a decrease (23.9%) (Berggren et al., 2023). This stands in contrast to findings from other countries (Australia, Bosnia and Herzegovina, Canada, China, the Czech Republic, Germany, Italy, Jordan, Poland, Saudi Arabia and the USA)), where declines in adolescent physical activity were observed regardless of country and continent as a result of implemented restrictions to contain the spread of the COVID-19 virus (Bozzola et al., 2023). The relatively stable levels in Sweden may be partly explained by the country´s open pandemic policy, where schools and sports associations remained open, adapting their activities instead of closing (Björkman et al., 2023). Nevertheless, the current interview findings also indicated differences in opportunities for leisure time physical activity, where youth in socioeconomically disadvantaged areas described that they did not participate in organized activities to the same extent as youth in more affluent areas during the pandemic. Youths also indicated that these differences had increased after the pandemic, mainly due to increased activity costs in combination with long-term parental unemployment during the pandemic. Results from the Swedish birth-cohort (Berggren et al., 2023), also showed a negative association between maternal educational level and physical activity during the pandemic (Berggren et al., 2023). Furthermore, results from the birth-cohort showed that being active in a sports club during the pandemic was protective of physical activity, which adolescents with lower socioeconomic position were less likely to be (Berggren et al., 2023).

The findings also emphasize the need for schools to be more supportive of physical activity. This is also in line with previous reports where the important role of schools is brought up (Andermo et al., 2020; van Sluijs Emf et al., 2021; World Health Organization, 2022). In Sweden, the school setting contributes to approximately one third (35%) of children and adolescents’ weekly physical activity on a moderate-to-vigorous intensity level (The Public Health Agency of Sweden, 2024b). Still, youths in the current study experienced spending a lot of time sitting and expressed a desire for more activity and natural movement during school days. Among other things, youths suggested the need for educating teachers about the benefits of physical activity for learning, and schools supporting and enabling teachers to implement more active classes. Swedish curricula state that “The school must also strive to offer all students daily physical activity within the framework of the entire school day”, and the Swedish National Agency for Education provides information and strategies (The Swedish National Agency for Education, 2024) on how schools and teachers can be more supportive of physical activity. However, there is no policy for actively implementing such strategies (The Ministry of Social Affairs, 2024). In relation to this, results from a study conducted in Swedish preschools showed that, in preschools with a policy for physical activity, children had more daily moderate-to-vigorous physical activity and were less physically inactive compared to preschools with no policy (Center for Epidemiology and Community Medicine, Region Stockholm, 2024). Moreover, a recent Swedish Government official report (SoU 2023:29) (The Ministry of Social Affairs, 2024) proposed that “All students must be given the opportunity and be encouraged to participate in health-promoting physical activities during the school day”. The report also concludes that clearer writing in school curricula is needed to strengthen students’ right to equal education and equal health (The Ministry of Social Affairs, 2024). Thus, although tips and guidelines are important, there is a need to complement these with policies that regulate physical activity in the school setting.

The findings also underline the interplay of social belonging, background and social norms for youth to be physically active. Inclusive spaces like football fields and youth centres enhanced community feelings and encouraged physical activity. However, there were also barriers in the form of performance and gender norms coupled with discrimination in the form of racialization and weight stigmatization. Research has shed light on the critical role of social norms shaping behaviour among adolescents (Wang et al., 2019). Kemp et al., (2022) further underscore the challenge posed by performance and gender norms in maintaining inclusive physical activity participation during adolescence. They propose a reframing of physical activity to better fit the needs of adolescents, as a way to counteract these norms and promote sustained engagement (Kemp et al., 2022). Moreover, Levi *et al*. (2024) conclude that community, environment and social norms support physical activity performance and should be integral components of interventions aiming at increasing physical activity among youth.

This study also sheds light on the balance between independence and safety in relation to engaging in physical activity. The findings underscore the crucial role of neighbourhood safety, which according to Nichol *et al*. (2010) has a more profound impact on youth physical activity than the mere presence of recreational facilities (Nichol et al., 2010). A noteworthy observation from our study is the heightened sense of outdoor risk reported by girls, leading to a reluctance to participate in physical activities. This aligns with the findings of Evenson et al., (2007) who established a correlation between girl´s perception of outdoor safety—encompassing factors such as crime rate, availability of streetlights, and visibility of other pedestrians—and the level of physical activity participation. The implications of these findings are significant. As Nichol et al., (2010) suggest, enhancing neighbourhood safety and improving individual perceptions of safety could be potent strategies for promoting physical activity among youth. Furthermore, these measures could potentially foster greater social interactions and community cohesion, thereby creating an environment that encourages physical activity.

In Sweden, only 2 out of 10 children meet the physical activity recommendations, with adolescents, and especially adolescent girls, being the most sedentary (Generation PEP Sweden, 2024). In the current study, girls expressed feeling less prioritized compared to their male peers in terms of their needs and preferences of physical activities in the school setting and discussed not being able to fully engage in physical education classes due to this. In a recent systematic review, which concluded a steady decline in adolescent girls’ physical activity globally (Duffey et al., 2021), the WHO highlights that gender-biased physical education curriculums in schools are a barrier, and suggests a shift from competitive sports to inclusive, non-skill-based activities to reverse the declining physical activity trend among adolescent girls (Duffey et al., 2021). They further emphasize the need for gender-responsive approaches and policies and recommend that future efforts should consider girls’ preferences to encourage their participation (Duffey et al., 2021).

### Strengths and limitations

This study focused exclusively on interviews with youth to foreground their voices and lived experiences, which are often marginalized in research and decision-making processes that directly impact them. This approach is grounded in a child rights-based perspective, recognizing young people as experts in their own lives (Lundy, 2007). A strength of the study is the inclusion of youth from both urban and rural settings, as well as youth from different socioeconomic areas. Future research may benefit from incorporating the perspectives of parents or other stakeholders to deepen understanding and broaden contextual insights. Language was not a limitation; all participating youths except for one spoke Swedish well enough to participate in the discussions. A possible limitation would be that most students were 14–15 years of age, and that additional older students (16–18 years) could have been included. Furthermore, although youth from both urban and rural settings were included, additional focus groups could have been conducted in rural settings as most of the youth were now from urban settings. Nevertheless, the inclusion of youths with various socioeconomic backgrounds enabled collection of diverse and rich data that contributed to the credibility of the findings and provided insights into youths’ current opportunities for physical activity as well as how these opportunities differed. Credibility was also endorsed through continuous discussions and interpretation of the findings between the authors. Inclusion of quotations increased transparency and further strengthened the credibility of the findings. Finally, the COREQ-checklist (Tong et al., 2007) was used to ensure inclusion of all necessary approaches.

### Conclusions and implications

The study provides valuable insights into the experiences and opportunities for physical activity among Swedish adolescents during and after the COVID-19 pandemic. The findings show that many Swedish youths managed to remain physically active during the COVID-19 pandemic. However, socioeconomic differences and perceptions of community safety affected opportunities for participation in organized sports and activities, both during and after the pandemic. The findings also underscore the important role of schools and the impact of gender and performance norms on physical activity, as well as young girls’ desire for “safe spaces” to focus on their training and development.

To create equal opportunities for physical activity going forward, it is recommended that governmental resources be allocated to support youth participation in organized activities in socioeconomically disadvantaged communities, and to enhance community safety. The findings also highlight the urgency of developing national policies that regulate and support physical activity in schools. This aligns with the conclusions of the Swedish Governmental report (SoU 2023:29) The Ministry of Social Affairs, (2024), which identifies schools as a key setting for increasing physical activity.

Moreover, schools need to challenge current gender and performance norms by re-evaluating the types of sports and activities scheduled during physical education classes. This is crucial for increasing students’, and especially young girls’, motivation and willingness to participate. Additionally, developing national and regional policies to support schools and teachers in promoting physical activity through active lessons, active breaks, and additional physical education classes is essential. These policies should focus on challenging gender and performance norms to motivate all students, particularly adolescent girls, to be more physically active.

While social belonging is essential for promoting physical activity, it is equally important to address the barriers that impede its positive impact. This necessitates further research and discussion to explore effective strategies for fostering inclusivity in physical activity.

## Ethics approval and consent to participate

The study was approved by the Swedish Ethical Review Authority (ref no. 2023–01085–01). Written informed consent was collected from all participating students prior to the focus group interviews.

## Data available statement

The datasets used and/or analysed during the current study are not publicly available due to current legislation provided by the ethical review board.

## Supplementary Material

Supplementary file 1_Interview guide.pdf
