# Supplementary material for: Unravelling the roadblocks and pathways to adolescents’ physical activity
Source: Int J Qual Stud Health Well-being. 2025 Jul 1;20(1):2524270. doi: 10.1080/17482631.2025.2524270 (PMC12217099; doi:10.1080/17482631.2025.2524270)
Supplement: Supplementary file 1_Interview guide.pdf [file ZQHW_A_2524270_SM7759.pdf]

Interview guide

What does physical activity mean to you?

What activities do you engage in that involve movement?

Can you tell me more about them?

Where are you usually active (indoors/outdoors)?

Does the physical environment/your surroundings matter? Can you describe/elaborate/give examples?

Are there any activities you would like to do that you don't do today?

Can you tell me more? For example, what are the reasons you can't do this, what would be required/what support is needed to make this possible?

How do you experience physical activity, what is good and what is less good?

Can you tell me more/give examples/elaborate?

Can you tell me what physical activity and leisure activities looked like during the pandemic?

Can you tell me more/give examples/elaborate?

Can you give examples of what you did during the pandemic?

Was there anything that made it difficult to be physically active during the pandemic?

Was there anything that made physical activity/activities easier during the pandemic?

Can you give examples of what you did instead of the activities that were canceled? (if relevant to ask)

What do you know about societal norms?

Are there any norms that limit young people's opportunities for physical activity?

Is there anything you haven't already mentioned that could make it easier for you or motivate you to be more physically active?

Can you tell me more? For example, what increases your motivation for physical activity, what support do you need?

Is there anything you haven't already mentioned that hinders or makes it difficult for you to be physically active?

Can you tell me more? For example, lack of time, demands from school, other things that make this a lower priority.

Do you have any activities that involve being sedentary for longer periods?

How do you experience being sedentary?

Can you tell me more/give examples/elaborate?

If you wanted to change the amount of sedentary time, how could you change it?

Can you tell me more? For example, what support do you need?
